# Anomia: Deciphering Functional Neuroanatomy in Primary Progressive Aphasia Variants

**DOI:** 10.3390/brainsci13121703

**Published:** 2023-12-11

**Authors:** Diliara R. Akhmadullina, Rodion N. Konovalov, Yulia A. Shpilyukova, Ekaterina Yu. Fedotova, Sergey N. Illarioshkin

**Affiliations:** Research Center of Neurology, 125367 Moscow, Russia; akhmadullinadr1@gmail.com (D.R.A.);

**Keywords:** primary progressive aphasia, anomia, voxel-based morphometry, functional connectivity

## Abstract

Naming decline is one of the most common symptoms of primary progressive aphasia (PPA). Most studies on anomia in PPA are performed without taking into account PPA variants, especially for action naming. Only limited data are available for the neuroanatomical basis of anomia considering differences in the pathogenesis of PPAs. The aim of our study is to investigate the associations between anomia severity for both noun and verb naming and gray matter (GM) atrophy, as well as accompanying functional connectivity (FC) changes in three PPA variants. A total of 17 patients with non-fluent (nfvPPA), 11 with semantic (svPPA), and 9 with logopenic (lvPPA) PPA variants were included in the study and underwent cognitive/naming assessments and brain MRIs. Voxel-based morphometry was performed to evaluate GM volume. A resting-state functional MRI was applied to investigate FC changes in the identified GM areas. The study shows that different brain regions are involved in naming decline in each PPA variant with a predominantly temporal lobe involvement in svPPA, parietal lobe involvement in lvPPA, and frontal lobe involvement in nfvPPA. Separate data for object and action naming in PPA variants are provided. The obtained results mainly correspond to the current understanding of language processing and indicate that the evaluation of language impairments is preferable for each PPA variant separately. A further analysis of larger cohorts of patients is necessary to confirm these preliminary results.

## 1. Introduction

Primary progressive aphasia (PPA) is a rare neurodegenerative disease characterized by pronounced clinical, genetic, and pathomorphological heterogeneities and predominant language impairment with the relative preservation of other cognitive functions [1]. Depending on the clinical symptoms and atrophy patterns, three PPA variants are distinguished [2]. Non fluent (nfvPPA) and semantic (svPPA) variants more often have underlying frontotemporal lobar degeneration pathology, usually tau and TDP-43, respectively, while logopenic-variant PPA (lvPPA), in most cases, has an Alzheimer’s disease pathology [3]. However, this division is not strict, since various pathomorphological and genetic variants can be observed in each PPA variant. In addition, PPA syndrome, usually nfvPPA, can develop as part of other neurodegenerative diseases, such as progressive supranuclear palsy or corticobasal syndrome, or manifest in combination with motor neuron diseases [4,5]. Such overlapping heterogeneity greatly complicates the diagnosis in clinical practice and the research in this field.

Clinically, nfvPPA is characterized by the apraxia of speech and/or agrammatisms and is associated with the left-sided atrophy of posterior frontal areas and the insula. Key features of svPPA are a loss of semantic knowledge about objects along with anomia and the predominantly left-sided degeneration of anterior temporal regions. LvPPA manifests as impaired repetition and single-word retrieval and is accompanied by the atrophy of the left posterior perisylvian and parietal regions. Over time, other symptoms may occur, including non-speech cognitive impairment or behavioral or movement disorders, but speech impairment remains the most prominent problem [1].

Naming difficulties are one of the most common PPA symptoms and can occur in each of the variants [6], while presenting different pathogeneses and corresponding manifestations. In svPPA, anomia develops as a result of semantic disorders [1]. In lvPPA, short-term phonological memory is impaired, and despite semantic knowledge being preserved, it cannot be converted into productive speech [1]. For nfvPPA, impaired naming is less typical but can be developed in the later stages or can be a secondary symptom caused by the severe apraxia of speech [7].

Given the slow progression and focal patterns of atrophy, PPA is a unique model that can be used to study the neuroanatomical foundations of speech and the pathogenesis of various symptoms, including anomia.

Several studies have previously explored the existing associations between gray matter (GM) brain volume and naming impairment in PPA. One of the first studies including a frontotemporal dementia group with 15 PPA patients showed that picture naming was associated with the GM atrophy of the left anterior lateral temporal, dorsolateral, and superior frontal cortices, superior parietal lobule, and striatum, as well as corresponding areas in the right hemisphere [8]. Other studies focusing on PPA show more focal results, mainly including various temporal regions and less frequently occurring frontoparietal areas [9,10,11,12,13,14,15,16,17]. However, these results were obtained predominantly for the general PPA group without taking into account the major differences in anomia pathogenesis among the PPA variants. Only a limited number of published studies focused on studying PPA variants separately. Two works on svPPA emphasized the role of the temporal lobe, especially its anterior parts, in object naming in this group [14,15]. A study by Migliaccio R. and colleagues included all three PPA variants and showed that picture naming was associated with the temporal pole volume in svPPA and the posterior and inferior temporal regions in lvPPA, with no correlations found for nfvPPA [7]. This work also demonstrated that the same analysis performed on the whole PPA group presented different results. Another limitation that is common in these studies is the deliberate restriction of the studied areas to the frontotemporal or frontotemporoparietal regions of the left hemisphere, which can potentially lead to false-negative results while increasing the statistical significance [7,9,11].

The main focus of the listed works was object naming, however action naming is also of particular interest. The existing data suggest that object and action naming may have a distinct neuroanatomical basis and can be damaged independently, although it remains a matter of debate. This theory is supported by the object–action naming dissociation that is typical for PPA with greater difficulty in action naming in nfvPPA patients and the opposite pattern of predominant object naming difficulty in svPPA patients [6,18]. The reason for this dissociation may be the pathogenesis differences among PPA variants mentioned above. Several studies have assessed action naming in PPA variants with various results [11,19,20,21]. All mentioned works studied PPA as a general group without a separate analysis of its variants. To our knowledge, no studies assessing action naming in PPA variants have been published to date.

The clinical manifestations of PPA variants may vary in different populations due to linguistic features, which in turn may be accompanied by differences in the underlying GM degeneration [22,23]. Previous cross-linguistic studies show that the naming function mainly depends on the same properties across all languages, but slight differences exist in naming performance [24,25]. There are no works that examine anomia in Russian native speakers with PPA to date.

The aim of this work is to identify anatomical correlations of anomia in each of the PPA variants in the Russian population and to compare the obtained data with previous studies and our current understanding of language pathophysiology. Here, we present our preliminary results.

## 2. Materials and Methods

The study was conducted at the Research Center of Neurology (Moscow, Russia). Local Ethical Committee approval was received and all participants signed an informed consent form. A PPA diagnosis was established in accordance with the current diagnostic criteria [2]. Participants were excluded from the study if they had any contraindications for MRIs, any conditions that could potentially lead to additional cognitive impairments (such as vitamin B12 or folic acid deficiencies, thyroid dysfunction, syphilis, etc.), or if any non-neurodegenerative structural brain changes were identified during the structural brain MRI scan (including post stroke lesions, brain tumor, hydrocephalus, severe white matter hyperintensity (Fazekas grades 2 and 3), etc.).

Thirty-seven patients with PPA diagnoses were enrolled in the study. They formed 3 groups according to the variant of the disease: 17 patients had nfvPPA, 11 patients had svPPA, and 9 patients had lvPPA. The demographic and clinical data of the participants are summarized in Table 1. No significant differences were found for gender, age, education, and disease duration between the groups.

Naming was assessed using the Tsvetkova language assessment scale [26]. The patients were presented with simple black and white drawings with a task to name the objects or actions depicted in them. For each correct answer, 1 point was provided, with the highest score being 60 points (30 points for object naming and 30 points for action naming). Mild articulation difficulties did not affect the score. If the right answer was provided after receiving a semantic clue or consisted of a phrase containing the required word, it was rated as 0.5 points. The same score was also provided in the case of single literal paraphasias. Naming impairments were also assessed using the Progressive Aphasia Severity Scale (PASS). This scale offers an evaluation of symptom severity ranging from 0 to 3 points (including a score of 0.5), where 0 means no difficulties are present and 3 signifies a severe impairment. We assessed the PASS «Word Retrieval and Expression» score for all groups and the «Articulation» and «Syntax and Grammar» scores for the nfvPPA group to evaluate their possible contributions to object- and action-naming impairments. The modified Addenbrooke’s cognitive examination III (ACE III) was used to assess global cognitive functions. The scale includes five cognitive domains—attention, memory, language, verbal fluency, and visuospatial abilities. The maximum total score is 100, with higher scores indicating better cognitive functioning outcomes.

Correlations between GM atrophy and naming performance in the PPA variants were assessed using voxel-based morphometry (VBM). The patients had brain MRIs on 3.0 T scanners (Magnetom Verio, Siemens; Magnetom Prisma, Siemens) with the acquisition of three-dimensional T1-weighted scans. Image pre-processing and a statistical analysis were performed using Statistical Parametric Mapping 12 software (Institute of Neurology; London, UK) running on MatlabR2020b (Mathworks; Natick, MA, USA). Pre-processing included the segmentation of scans into GM, white matter, and cerebrospinal fluid using the DARTEL (Diffeomorphic Anatomical Registration Through Exponentiated Lie Algebra) algorithm, the normalization of the resulting images in the Montreal Neurological Institute (MNI) space, and further smoothing with an 8 mm full-width at half-maximum (FWHM) Gaussian kernel. To find the possible associations between the anomia severity measured by language assessment scales and GM volume, a multiple regression was performed, adjusting for gender, age, and intracranial volume, calculated as the sum of GM, white matter, and cerebrospinal fluid volumes. Additionally, a separate analysis was performed to assess the correlations between action and object naming. The analysis was limited to the cerebral cortex. Only clusters with a volume >50 voxels were included in the results. Due to the relatively small sample size of the groups, the significance level was set at *p* < 0.001, uncorrected for multiple comparisons. A graphical presentation of the VBM results was created using bspmview v.20161108 software [27].

In the second part of the study, we measured the functional connectivity (FC) between the identified VBM areas associated with anomia and other brain regions (seed-to-voxel analysis) to identify FC changes that correlated with anomia severity in each of the PPA variants. Resting-state functional MRI (rs-fMRI) scans were performed using a T2-weighted echoplanar sequence (repetition time: 3000 ms, echo time: 30 ms, flip angle: 90°), recording 49 slices at a thickness of 2 mm. The participants were instructed to stay awake with their eyes closed and to not think of anything in particular during the scan. Image pre-processing, statistical analyses, and result outputs were performed using the CONN v.21a toolbox [28] running on MatlabR2020b (Mathworks; Natick, MA, USA). Post-processing of functional and anatomical data included a realignment with the correction of susceptibility distortion interactions, slice-timing correction, outlier detection, direct segmentation, MNI-space normalization, and smoothing with a Gaussian kernel with an 8 mm FWHM. Functional data were further denoised using a standard denoising pipeline [29] to exclude the potential confounding effects of white matter and cerebrospinal fluid timeseries and motion parameters followed by bandpass frequency filtering to remove BOLD timeseries results below 0.01 Hz or above 0.1 Hz. For each of the PPA variants, a seed-to-voxel analysis was performed using a multiple regression, with age and gender as the covariates, to identify the correlations of FC changes with confrontation-naming impairment measured by language assessment scales. The results were thresholded using a combination of a *p* < 0.001 voxel-level threshold, and *p* < 0.05 corrected for multiple comparisons (False Discovery Rate [FDR]) cluster-size threshold.

A statistical analysis was performed using the SPSS Statistics 26.0 software package (IBM, Armonk, NY, USA). Differences between nominal and ordinal variables were compared between groups using the Fisher’s exact test and between quantitative variables using the Kruskal–Wallis test with the Bonferroni correction for multiple comparisons. A linear regression analysis was used to predict relationships between the variables. A statistical significance was indicated if *p* < 0.05.

## 3. Results

The cognitive examination results are presented in Table 2. ACE III revealed that there was a more pronounced cognitive decline in the svPPA group compared to the nfvPPA group, with total score median values of 38 and 71, respectively. According to the PASS evaluation, cases with minimal or no anomia prevailed in the nfvPPA group, while mild and moderate naming impairments were more common in other groups. However, the differences were not statistically significant. The naming examination assessed by the Tsvetkova language assessment scale revealed that the most pronounced anomia in both object and action naming was observed in the svPPA group, where the total score was less than 30 in more than 75% of the cases. Significant impairments were also observed in the lvPPA group, where the median total score was 34 points out of 60. In the nfvPPA group, anomia severity was milder (median total score: 55 points) with a statistically significant difference between this group and the svPPA group. Action-naming impairment prevailed in the nfvPPA group, while in the svPPA group, object naming was more affected. In the lvPPA group, the difference was less pronounced with almost the same scores.

**Figure 1:**
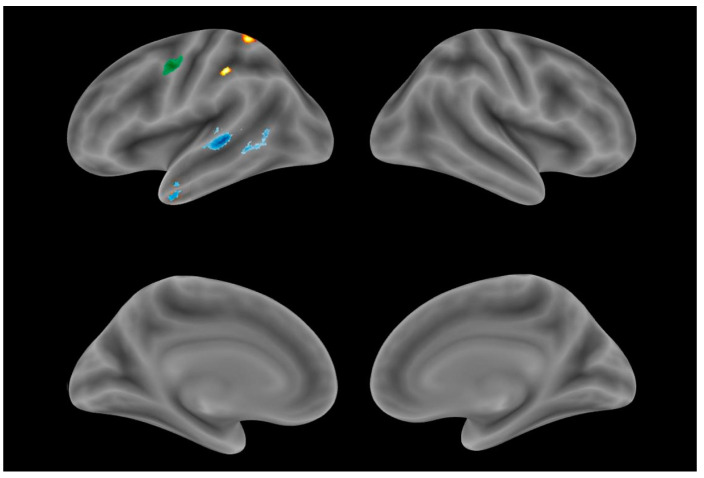
Identified gray matter areas correlating with naming performance in each PPA variant: nfvPPA (green), svPPA (blue), lvPPA (yellow).

The VBM revealed the brain regions where atrophy was associated with anomia in each of the PPA variants (Table 3, Figure 1). In the svPPA group, correlations were observed, with the atrophy of the left temporal pole (TP) and posterior parts of the superior (STG) and middle temporal gyri (MTG). In the lvPPA group, naming impairment was correlated with the GM volume of the left parietal lobe, namely, the supramarginal gyrus and superior parietal lobule. In the nfvPPA group, associations were found with the GM degeneration of the left precentral gyrus. In addition to this finding for the nfvPPA group, we conducted a linear regression analysis of naming performance (a dependent variable) and articulation and syntax/grammar impairments (independent variables). The articulation score (apraxia of speech and/or dysarthria) predicted 42.8% of the naming performance score for nfvPPA patients in contrast to syntax/grammar impairments, which did not significantly contribute to the naming performance.

**Figure 2:**
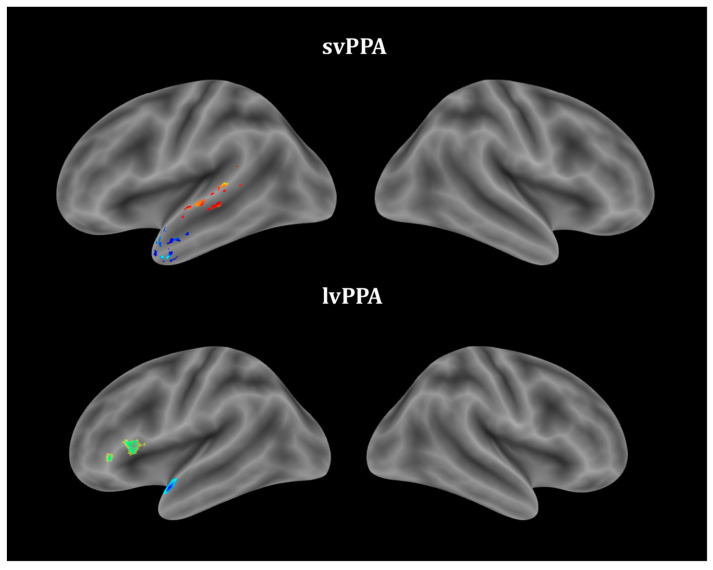
Gray matter regions associated with object- and action-naming performances in PPA variants: object naming (blue), action naming (yellow–red), both (green).

Correlations between the GM volume and object and action naming were assessed separately for each PPA variant (Table 3, Figure 2). No significant correlations were observed in the nfvPPA group. Action naming was associated with the GM atrophy of the left mid-posterior parts of the STG and MTG in the svPPA group and IFG atrophy in the lvPPA group. More severe object naming correlated with left temporal pole atrophy in the svPPA group and both left TP and IFG atrophy in the lvPPA group.

**Figure 3:**
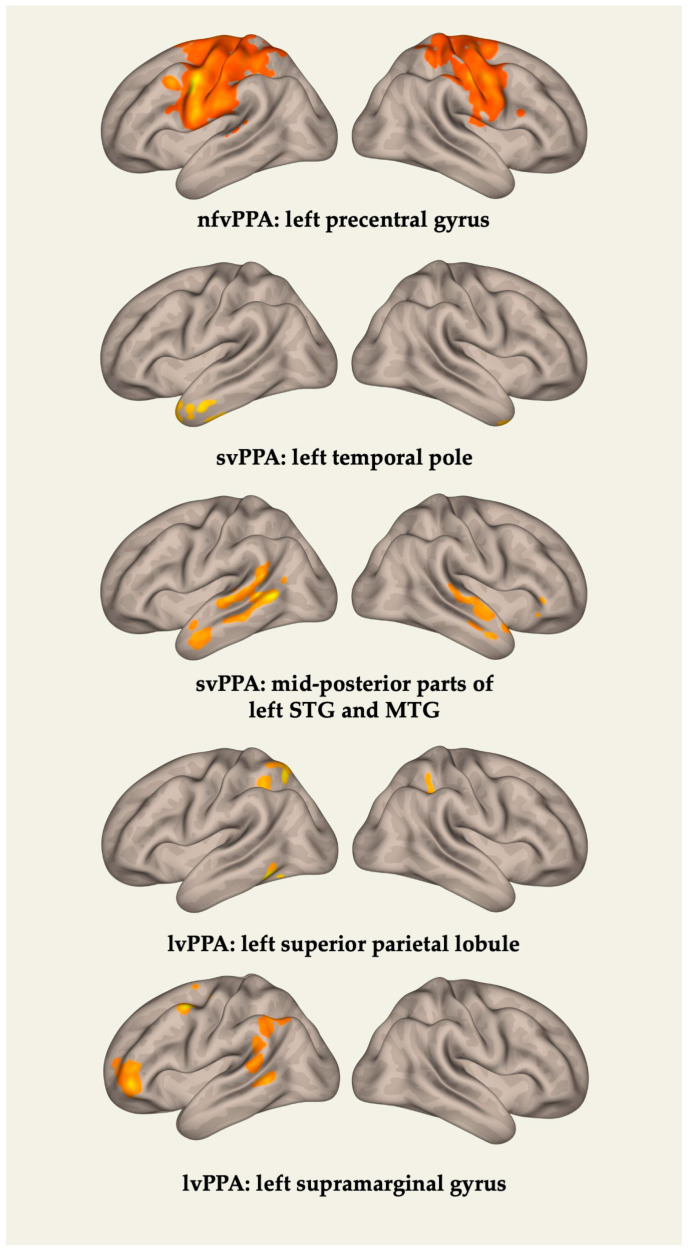
Functional connectivity changes associated with naming performance in PPA variants.

Then, for each of the PPA variants, FC changes between the areas identified by VBM and the rest of the GM regions were assessed. The results are presented in Table 4 and Figure 3. In the nfvPPA group, anomia severity was associated with an FC reduction between the left precentral gyrus and the postcentral gyri, supplemental motor area (SMA), premotor cortices on both sides, and the right precentral gyrus. In the svPPA group, correlations were identified with an FC decrease between the left TP and the left parahippocampal and fusiform gyri, anterior parts of the MTG and inferior temporal gyri, the hippocampus, and the right TP. FC changes in the posterior parts of the STG and MTG were assessed separately. Associations were found with an FC disruption between this cluster and the regions of the temporoparietal junction and TP of the left hemisphere, as well as the STG and TP of the right hemisphere. In the lvPPA group, naming impairment correlated with an FC reduction between the superior parietal lobule and the left inferior parietal lobule, the supramarginal gyrus on both sides, as well as between the left supramarginal gyrus and the dorsolateral prefrontal cortex (DLPFC), the premotor cortex, the angular gyrus, and the MTG of the left hemisphere.

## 4. Discussion

In the study, we found brain regions associated with anomia in PPA. The identified areas were localized exclusively in the left hemisphere of the brain and were different for each of the PPA variants, not coinciding with each other. The latter highlights that a separate study of PPA variants is more preferable than combining all the cases in one group.

Naming difficulties in most of the nfvPPA cases (70.6%) were absent or of a very mild severity, and the VBM revealed their association with precentral gyrus atrophy. This finding and the results of the linear regression indicate that naming difficulties in our nfvPPA group are most likely secondary symptom due to the apraxia of speech (observed in all cases) and/or dysarthria due to concomitant motor neuron disease (observed in 17.6% of cases). This assumption was also supported by the rs-fMRI data, according to which anomia is associated with an FC disruption between the left precentral gyrus and the premotor cortex and SMA, both of which play an important role in the development of the apraxia of speech [30,31]. No associations were observed in the nfvPPA group when noun and verb naming were examined separately. Most likely, this was due to the relative preservation of both functions and a lower value variance in comparison with the total score.

The most pronounced anomia was observed in svPPA and lvPPA patients. The regression analysis showed that, in the svPPA group, anomia was associated with the atrophy of the left TP and the mid-posterior parts of the STG and MTG. A separate evaluation revealed that object and action naming in the svPPA group was associated with the left temporal pole and left mid-posterior temporal regions, respectively, with no overlap between the areas. These results are consistent with our current understanding of speech neuroanatomy and the svPPA clinical picture. As previously mentioned, the main svPPA symptom was the loss of semantic knowledge about objects, which predominantly led to semantic errors in naming [2]. The left TP is one of the core atrophy areas in svPPA patients and, according to the recent research, plays an important role in storing semantic knowledge [32,33]. Separate studies have also shown an association between left TP volume and naming both in svPPA patients and in the general PPA group [7,9,10,14]. Moreover, similar results were obtained in a study on post-stroke aphasias, where the presence of semantic errors in naming correlated, among other things, with damage to the left TP and adjacent areas [34]. Less typical for svPPA patients was the identified association between naming difficulties and the atrophy of the left mid-posterior temporal lobe. At the same time, some works show that damage to this area can play a certain role in the pathogenesis of anomia in svPPA patients and is associated primarily with the loss of conceptual knowledge about objects and with the complete inability to respond to the presented stimulus [14]. In the previously mentioned study on post-stroke aphasias, damage to the mid-lateral temporal lobes was also associated with semantic naming errors [34]. According to the current dual-stream model theory of speech and language processing, this area belongs to the ventral pathway and is involved in tasks that require access to phonological information, including naming, as well as the active storage of phonemic information [35]. Thus, despite the fact that this finding is not typical for svPPA, it is consistent with current ideas about the functional anatomy of language. The rs-fMRI results are also consistent with the dual-stream model theory. The FC reduction in svPPA is mainly observed in the ventral pathway structures (left and right TPs and adjacent parts of the temporal lobes, the fusiform gyrus, and STG) that are responsible for semantic knowledge and speech perception, as well as between the ventral and dorsal pathways (the middle parts of the STG and MTG with their posterior parts and temporoparietal junction). It is of interest that lesions of the most posterior parts of the temporal lobe are more often associated with lvPPA and, predominantly, lexical mistakes made during confrontational naming, which are typical for this variant [7,33]. It is possible that our results reflect disease progression with GM degeneration spreading to the more posterior temporal regions than normally seen at the beginning stages of svPPA and the emergence of additional lexical impairments. This assumption was also indirectly confirmed by the FC reduction between the posterior regions of the temporal lobes and the temporoparietal junction, as the degeneration of the latter is mostly observed in lvPPA patients [2]. Apart from overall naming, the mid-posterior temporal cortex was also associated with action naming deterioration in svPPA patients. This finding is consistent with some previous reports on verb naming in PPA and neurodegenerative disorders [19,21], as well as lesion-symptom mapping studies [36,37]. A number of fMRI studies have also previously shown that posterior MTG is activated during action word processing and both action and non-action verb naming [38,39,40].

Compared to svPPA, lvPPA has a different anomia pathogenesis with damage to the lexical and the preservation of semantic component, as well as the presence of phonological errors [2,33]. Consistent with this theory, the VBM revealed that, in contrast to svPPA, overall anomia in lvPPA correlated with the GM volume in other brain regions, namely, the supramarginal gyrus and superior parietal lobule. Supramarginal gyrus atrophy is typical for lvPPA and, according to the numerous data, is one of the core areas of language processing. Most studies agree that it belongs to the dorsal pathway of the dual-stream model, being responsible for the lexical component of naming and providing a connection with motor cortex areas [41]. For example, Schwartz et al. showed that stroke lesions in this area caused phonological errors in naming. They suggested that supramarginal gyrus damage is associated with the disruption of the selection or short-term buffering of phonological units [34]. In addition, it has been stated that post-stroke lesions of the supramarginal gyrus lead to the development of conduction aphasia, which somewhat resembles the clinical picture of lvPPA, including impaired repetition and the presence of phonological paraphasia [42,43,44]. Moreover, the association of supramarginal gyrus atrophy with anomia was previously shown in the general PPA group, appearing at later stages of the disease [9]. The rs-fMRI data show a correlation of anomia with an FC reduction between the supramarginal gyrus and other dorsal pathway structures, such as the angular gyrus and the posterior parts of the MTG, with functions similar to the supramarginal region, as well as with the premotor cortex and DLPFC, which are responsible for the motor component of speech production. In contrast to the supramarginal gyrus, the role of the superior parietal lobule in language implementation remains ambiguous. It is believed that this area is not included in the ventral or dorsal pathways and does not play any role in auditory or somatosensory feedback. However, it participates in maintaining visual attention, executive functions, and working memory [45,46,47]. Thus, these results may reflect secondary picture-naming difficulties due to the impairment of one of the mentioned cognitive domains. Unlike in the svPPA patients, in the lvPPA patients, the left IFG volume correlated with both object and action naming. Although this finding corresponds to previous PPA studies and works on action naming [11,20,40,48], this area damage is not typical for lvPPA and is rarely associated with noun-naming impairments. On the contrary, the correlation of object naming with the left temporal pole is more readily expected and probably has the same underlying mechanism as svPPA. It can reflect disease progression and the emergence of additional semantic deficits.

Despite the limitations listed below, our results provide important insights into the neuroanatomical basis of naming in PPA, significantly expanding on the currently available information. The focus of our work was a separate study of PPA variants for a more precise assessment of naming impairment. We showed that anomia correlated with different brain regions in each of the variants, and these findings correspond to the difference in their pathogenesis. Some of the initial data for the anatomical basis of action versus object naming in different PPA variants were obtained and also differed between the groups. These results suggest that a separate analysis of PPA variants is more preferable and should be more prevalent in the future. GM atrophy data were supplemented by rs-fMRI results, which made it possible to create a more complete picture of anomia pathogenesis. This study was also the first study on naming in PPA in the Russian population. Our results indicate that there are no significant differences in comparison with the existing data obtained for other populations; however, further studies with direct comparisons are necessary to confirm this suggestion.

## 5. Limitations

It should be noted that our work had some limitations. One of them was the naming assessment methodology. For the quantitative evaluation, we used the Tsvetkova language assessment scale. However, this scale was mainly developed for post-stroke aphasia and had some disadvantages when used for patients with PPA. One of the main limitations was that the types of naming errors were not recorded, which could help in determining the type of anomia. Another disadvantage was the absence of pictures with more infrequently used items or actions, which did not allow us to detect some milder impairments. In addition, only semantic clues were incorporated in the naming evaluation when multiple choices and phonological clues are considered more effective for differentiating between PPA variants [1,6]. At the same time, it should be noted that this scale was chosen based on certain advantages. First of all, it was developed for native Russian speakers, taking into account its linguistic characteristics, while the other most popular scales for naming assessments have not been validated for the Russian population to date. Another important advantage was the presence of naming tasks for both objects and actions. Other limitations of our study included the relatively small groups, which was primarily due to the low PPA prevalence, the lack of a longitudinal assessments of anomia and associated neuroimaging changes, and the limitation of the study area to the cerebral cortex, which did not allow us to assess the involvement of the cerebellum, despite the current knowledge of its importance for cognitive functions. The latter was mainly due to small study groups and to increase the statistical significance and avoid possible false-negative results, and thus could be avoided by enrolling more participants in the study. These limitations must be taken into account when planning future research in this area.

## 6. Conclusions

The brain areas involved in anomia development in PPA variants and their functional connectivity with other brain regions were identified. Each PPA variant showed a different pattern of the brain regions involvement. Naming impairment in the svPPA group was associated with left temporal lobe atrophy and the disruption of FC primarily within the ventral pathway. In the lvPPA group anomia severity was associated with left parietal lobe degeneration and a loss of FC in the regions of the dorsal pathway. Naming difficulties in the nfvPPA group were either absent or of mild severity and, most likely, were secondary symptom due to the apraxia of speech/dysarthria and damage to the left precentral gyrus and its connections to other motor cortex areas. The revealed heterogeneity of the neuroanatomical basis of naming decline in PPA variants emphasizes the different pathogeneses of PPA symptoms and indicates that it is more preferable to study each PPA variant separately rather than in one group. The study had several limitations that encourage the replication of the obtained results in larger PPA cohorts with a longitudinal analysis.

## Figures and Tables

**Table 1:** Demographic and clinical characteristics of the study participants.

	nfvPPA (n = 17)	svPPA (n = 11)	lvPPA (n = 9)
Age, years ^1^	64 [60; 67]	67 [63.5; 68.5]	65 [56; 67]
Gender (m/f), n (%)	6/11 (35/65%)	5/6 (45/55%)	6/3 (67/33%)
Education, years ^1^	15 [13.5; 15]	15 [13; 15]	14 [13; 16]
Disease duration, months ^1^	48 [36; 60]	36 [16; 48]	36 [23; 48]

^1^—values are presented as Me [Q1; Q3]; m—male, f—female.

**Table 2:** Results of the cognitive assessment for the study participants.

	nfvPPA (n = 17)	svPPA (n = 11)	lvPPA (n = 9)
ACE III, total score/100 ^1^	71 [45; 83] *	38 [26; 50] *	53 [37; 75]
PASS: word retrieval and expression, n			
Normal (0)	5	1	0
Very mild impairment (0.5)	7	1	3
Mild impairment (1)	3	5	5
Moderate impairment (2)	2	4	1
Severe impairment (3)	0	0	0
Tsvetkova language assessment scale:			
- Naming, total score/60 ^1^	55 [40; 59] *	27.5 [15; 29.5] *	34 [20.75; 49.5]
- Object naming/30 1	29 [22; 29] *	11 [10; 16] *	20 [16; 26]
- Action naming/30 1	26 [18; 29] *	15 [4.5; 20] *	19 [11; 24]

^1^—values are presented as Me [Q1; Q3]; *—statistically significant difference between groups.

**Table 3:** Correlations between GM volume of cortical regions and naming performance in PPA variants.

Cortical Region	Cluster Size	T Value	MNI Coordinates (x, y, z)
nfvPPA: Tsvetkova language assessment scale, naming (total score)			
Left precentral gyrus	68	4.04	−46, 4, 48
		3.55	−44, 4, 40
svPPA: PASS, word retrieval and expression			
Left STG, MTG	683	17.76	−60, −57, 10
		11.53	−60, −62, 2
		11.20	−66, −51, 8
svPPA: Tsvetkova language assessment scale, naming (total score)			
Left temporal pole	96	6.62	−58, 8, −27
svPPA: Tsvetkova language assessment scale, action naming			
Mid-posterior parts of left STG, MTG	221	7.56	−66, −48, 12
svPPA: Tsvetkova language assessment scale, object naming			
Left temporal pole	175	10.06	−54, 16, −30
lvPPA: PASS, word retrieval and expression			
Left superior parietal lobule	88	8.48	−26, −46, 62
lvPPA: Tsvetkova language assessment scale, naming (total score)			
Left supramarginal gyrus	51	4.18	−45, −36, 36
lvPPA: Tsvetkova language assessment scale, action naming			
Left IFG, pars triangularis	85	7.32	−50, 42, 16
Left IFG, pars triangularis	99	6.26	−52, 45, 0
lvPPA: Tsvetkova language assessment scale, object naming			
Left IFG, pars triangularis	183	10.20	−50, 42, 0
Left temporal pole, superior part	55	6.13	−54, 15, −10

STG—superior temporal gyrus. MTG—middle temporal gyrus. IFG—inferior frontal gyrus.

**Table 4:** Correlations between FC changes in brain regions identified by VBM with naming performance in PPA variants.

Cortical Region	Cluster Size	MNI Coordinates (x, y, z)
nfvPPA: left precentral gyrus		
Left and right precentral gyri, postcentral gyri, SMA, superior and middle frontal gyri	16,729	−52, −2, 42
svPPA: left temporal pole		
Left parahippocampal and fusiform gyri, hippocampus, temporal pole	538	−18, 4, −34
Right temporal pole	244	22, 12, −44
Anterior parts of left MTG and inferior temporal gyrus	121	−58, −4, −36
svPPA: mid-posterior parts of left STG and MTG		
Left temporoparietal junction, posterior parts of STG and MTG	832	−60, −52, 6
Right STG	226	72, 20, −2
Left temporal pole, anterior parts of MTG	173	−52, 4, −30
Right temporal pole	167	60, 10, −22
lvPPA: left superior parietal lobule		
Left superior parietal lobule	287	−24, −66, 64
Left inferior parietal lobule	117	−32, −42, 46
Posterior part of inferior temporal gyrus	90	−54, −64, −12
Right supramarginal gyrus	77	38, −40, 42
lvPPA: left supramarginal gyrus		
Left DLPFC	460	−40, 44, −6
Left supramarginal and angular gyri	437	−52, −46, 34
Posterior part of left MTG	277	−64, −50, 6
Left premotor cortex	201	−36, 2, 60

SMA—supplementary motor area. STG—superior temporal gyrus. MTG—middle temporal gyrus. IFG—inferior frontal gyrus. DLPFC—dorsolateral prefrontal cortex.

## Data Availability

The data that support the findings of this study are available from the corresponding author upon reasonable request. The data are not publicly available due to privacy and ethical restrictions.
